# DNA Replication Errors Drive Genome‐Wide Small Inverted Triplication Dynamics

**DOI:** 10.1002/advs.202521949

**Published:** 2026-04-07

**Authors:** Yi Lei, Yu Zhou, Haitao Sun, Hang Yuan, Xinyu Pei, Jessica D. Hess, Yao Yan, Zunsong Hu, Mian Zhou, Zhaohui Gu, Li Zheng, Xiwei Wu, Binghui Shen

**Affiliations:** ^1^ Department of Cancer Genetics and Epigenetics Beckman Research Institute, City of Hope Duarte California USA; ^2^ Department of Computational and Quantitative Medicine Beckman Research Institute, City of Hope Duarte California USA

**Keywords:** cancer genomics, DNA replication, flap endonuclease 1 (FEN1), Okazaki fragment maturation (OFM), small inverted triplication (SIT)

## Abstract

Structural variants (SVs) have a profound impact on phenotype and diversity and are associated with human diseases. To explore the origination of SVs, we have analyzed 1,340 cancer genomes with annotation of 4,608 novel small inverted triplication (SIT) events and found that *FEN1* is strongly associated with SIT incidence. Then, we performed long‐read sequencing and developed PacBioR to annotate SITs in yeast *FEN1* mutant cells. We found that SIT structures mimic classic inverted triplications but with a smaller DUP/IN/DUP structure of 148/160/148 bp on average, with a spacer sequence of 30 bp and breakpoint junction of 6 bp. We showed that breakpoints of SITs preferentially occurred at nucleosome midpoints, aligned with Okazaki fragment termini. We further identified that SIT harbored in plasmids were precisely eliminated via DNA polymerase slippage over SIT‐derived hairpin structures in *E. coli* system. This study provides mechanistic insight into SIT origination and offers practical tools for future studies on genome rearrangements.

## Graphical abstract

This study provides insight into the dynamic equilibrium mechanism of a novel structural variant, small inverted triplication (SIT), which is generated by misalignment of the 3’ flap generated under DNA replication stress with palindromic sequence. Alternatively, the end sequence may fold back on itself to form an inverted fragment. SITs are partially repaired and dynamically maintained in vivo, via DNA polymerase slippage over the hairpin structure to the original loci.

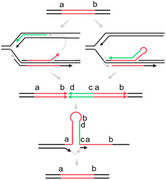

## 1 Introduction

Genome stability is essential for cell survival and normal growth but can be compromised by structural variants (SVs) such as insertions, deletions, inversions, and duplications [1, 2, 3]. Depending on the genomic context and affected genes, SVs are associated with many human diseases including, but not limited to, cancers [4]. One of the most important endeavors of the biomedical community is the discovery and characterization of SVs across human genomes [5].

Conventional technologies such as karyotyping [6], fluorescence in situ hybridization [7, 8], and comparative genomic hybridization arrays [9] have served as important methods for detecting and validating SVs. However, their limited sensitivity and resolution constrain the genome wide detection in proliferating cell populations and hinder the discovery of novel SVs. High‐throughput sequencing technologies have now become indispensable for investigating SVs at the whole‐genome level. Various short‐read‐based approaches for SV detection have been developed, which typically rely on strategies such as read‐depth analysis [10], discordant read‐pair mapping [11], split‐read alignment [12], local assembly [13], or combinations of these methods [14, 15, 16]. Although short‐read technologies have been widely applied in large‐scale projects, including the 1000 Genomes Project [17, 18], they are not ideal to detect and characterize SVs. In contrast, long‐read sequencing provides several advantages for SV detection [19, 20, 21, 22]. In particular, PacBio high‐fidelity (HiFi) sequencing generates long (10–25 Kbp) and highly accurate (> 99.9%) sequencing reads [23], making it well‐suited for characterizing SVs [24, 25]. However, until the current work, the identification of SVs has been challenging [26], particularly for rare and complex SVs.

One specific type of SVs, the inverted triplication, consists of an inverted central copy flanked by two direct copies (DUP/IN/DUP) [27], and was first identified using fluorescence in situ hybridization [28]. Since then, increasing numbers of locus‐specific inverted triplications have been reported, and accumulating evidence indicates that such structures can occur on nearly every human chromosome [27, 29, 30, 31, 32, 33, 34, 35, 36, 37, 38, 39]. To date, studies of individual inverted triplication cases have led to three proposed models for their formation [40, 41]. Brewer et al. observed that an inverted triplication is generated at the *SUL1* locus when the budding yeast *Saccharomyces cerevisiae* is grown long‐term in sulfate‐limiting chemostats and proposed the origin‐dependent inverted‐repeat amplification (ODIRA) model [41]. This model suggests that replication errors at pre‐existing, interrupted inverted repeats can generate extrachromosomal inverted dimeric intermediates, which can autonomously replicate and reintegrate into the genome, thereby forming an inverted triplication [27, 41, 42]. Carvalho et al. analyzed a disease‐specific cohort with an inverted triplication that occurred at the *MECP2* locus and proposed that these structures can arise through a combination of homology‐directed break‐induced replication (BIR) and template switching, or through microhomology‐mediated BIR and nonhomologous end joining [40]. In a subsequent study, they proposed an iterative template switching model, in which replication forks encountering inverted sequences undergo multiple rounds of template switching, ultimately producing an inverted triplication [29].

In the current study, we report a novel SV that shares structural features with the identified inverted triplications but is smaller in size than those previously described [27, 41, 42], with a median size of 148/160/148 bp (DUP/IN/DUP), which we characterize as a small inverted triplication (SIT) arising from DNA replication errors. Using 1,340 short‐read sequencing datasets, we identified the occurrence of SIT in different cancer genomes. Subsequent mutational analysis suggested that 4,002 genes with functional deficient mutations were significantly enriched in the high‐incidence SIT group, and *FEN1* was among the top 10 most significantly enriched genes. We performed long‐read sequencing of the *FEN1* yeast homologue (*rad27Δ*) mutant and 11 other strains, and developed PacBioR to detect SVs, including SITs, in the HiFi sequencing data. The results showed a marked increase in the incidence of SIT events in the *rad27Δ* mutant compared to the other strains. The breakpoints of SITs preferentially occurred at nucleosome midpoints and aligned with Okazaki fragment termini. Moreover, we found that SIT structures are inherently unstable within cells, and that knockout of *sbcC*, *sbcD*, or *rep* suppressed their elimination, whereas deletion of *dnaQ* or *holC* promoted such a loss. Taken together, our study provides the most comprehensive insights to date into the formation and elimination mechanisms of SITs.

## 2 Results

### 2.1 *FEN1* Mutations Exhibited a Striking Enrichment in the High SIT Incidence Cancer Population

**FIGURE 1:**
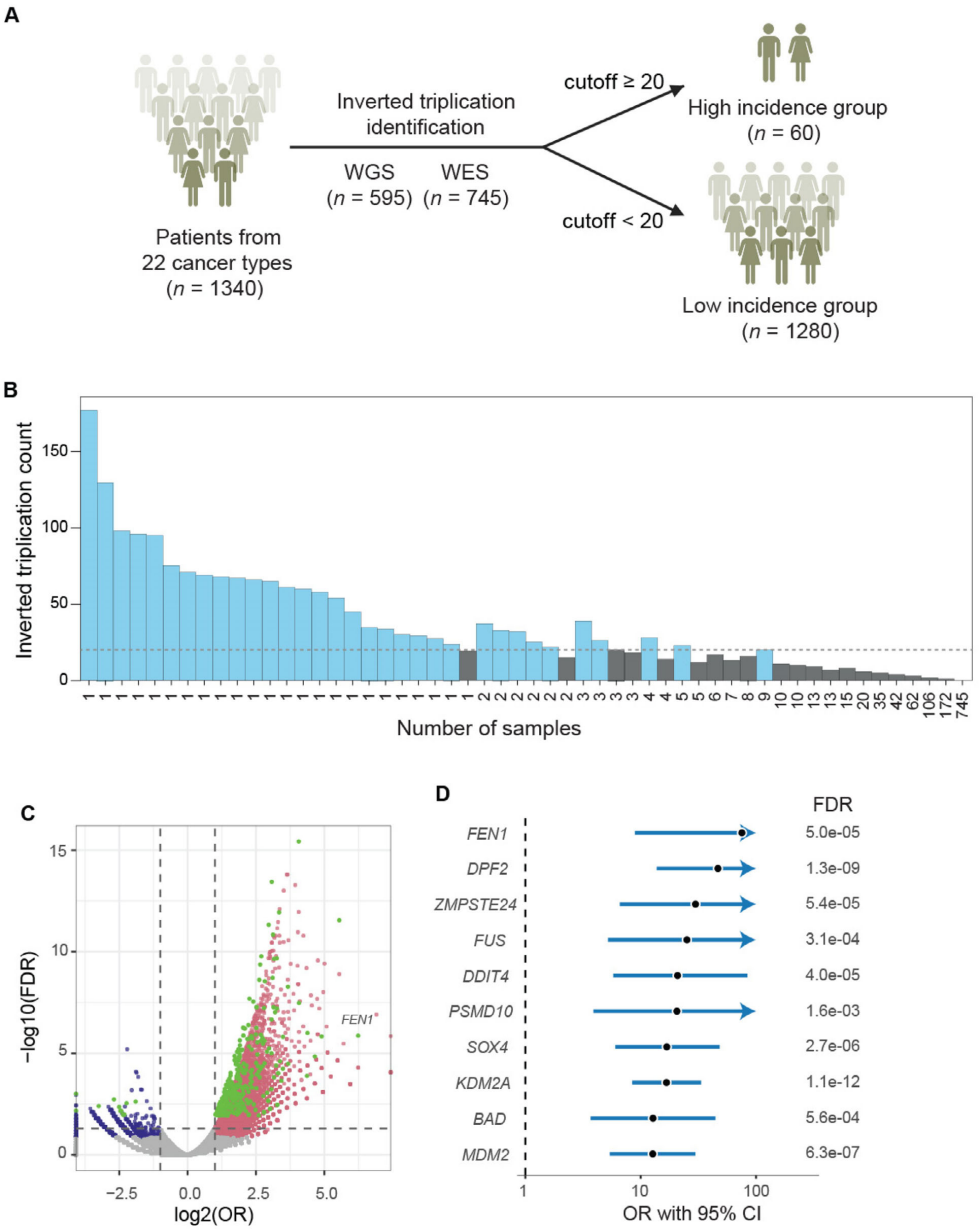
SIT incidence is shaped by individual genetic background. (A) A total of 1,340 samples spanning 22 cancer types were analyzed for SIT events detection using WGS and WES sequencing data. Based on the number of SITs detected, samples were classified into a high‐incidence group or a low‐incidence group. (B) Distribution of 4,608 SIT events across individual samples (Table S1). Each bar represents the number of samples with a given SIT count. The dashed line indicates the cutoff of 20 SITs per sample, corresponding to the 90th percentile of non‐zero SIT samples, which was used to define high‐ and low‐incidence groups. (C) Volcano plot showing genes significantly enriched for mutations in high‐ or low‐incident groups. The x‐axis represents the log2 odds ratio (log2OR) of mutation enrichment, and the y‐axis represents ‐log10 false discovery rate (FDR). Genes significantly enriched in the high‐incidence group (log2OR > 1, FDR <0.05) or low‐incidence group (log2OR <−1, FDR <0.05) were identified using Fisher's exact test. (D) Forest plot showing the top 10 DNA‐related genes with the highest OR for enrichment in the high‐incident SIT group. Dots indicate OR, horizontal lines represent 95% confidence intervals. Detailed results are provided in Table S3.

We first characterized SIT structures in B‐cell Acute Lymphoblastic Leukemia (B‐ALL) patients (Figure S1 and Table S1). To determine whether such structure could be detected in other cancers, we analyzed 1,340 short‐read sequencing datasets from patients representing 22 cancer types and identified 4,608 SIT events (Figure 1A, Tables S1 and S2, and Figure S1B,C). To explore the genetic basis underlying SIT formation, we performed a mutational analysis and identified 17,727 genes with functional deficient mutations. To evaluate the association of SIT events with gene mutations, we defined two groups: Samples with ≥ 20 SIT events, corresponding to 90th percentile of non‐zero cases, as group 1 (*n* = 60) and with zero events as group 2 (*n* = 745) (Figure 1B, Tables S1 and S2). Comparative analysis showed that a total of 4309 genes were significantly enriched (Fisher's exact test, Odds ratio (OR) > 2 and FDR <0.05), including 4,002 and 307 genes in groups 1 and 2, respectively (Figure 1C and Table S3). For the genes significantly enriched in group 1, the Gene Ontology (GO) analysis showed that the enriched mutant genes were involved in nucleic acid metabolism, such as RNA splicing, DNA replication, recombination, and repair (Figure S2 and Table S4). When we focused on the 1,179 DNA‐related genes identified by the GO and KEGG databases (Table S5), the results showed that 303 and 11 genes were enriched in group 1 and group 2, respectively (Figure 1C and Table S5). The top 10 genes with the highest OR value are shown in Figure 1D. Among them, *FEN1* mutations exhibited a striking enrichment in the high SIT incidence group (Figure 1D and Table S3).

### 2.2 PacBioR: A High‐Performance Tool for Accurate Annotation of SITs with HiFi Sequencing Data

**FIGURE 2:**
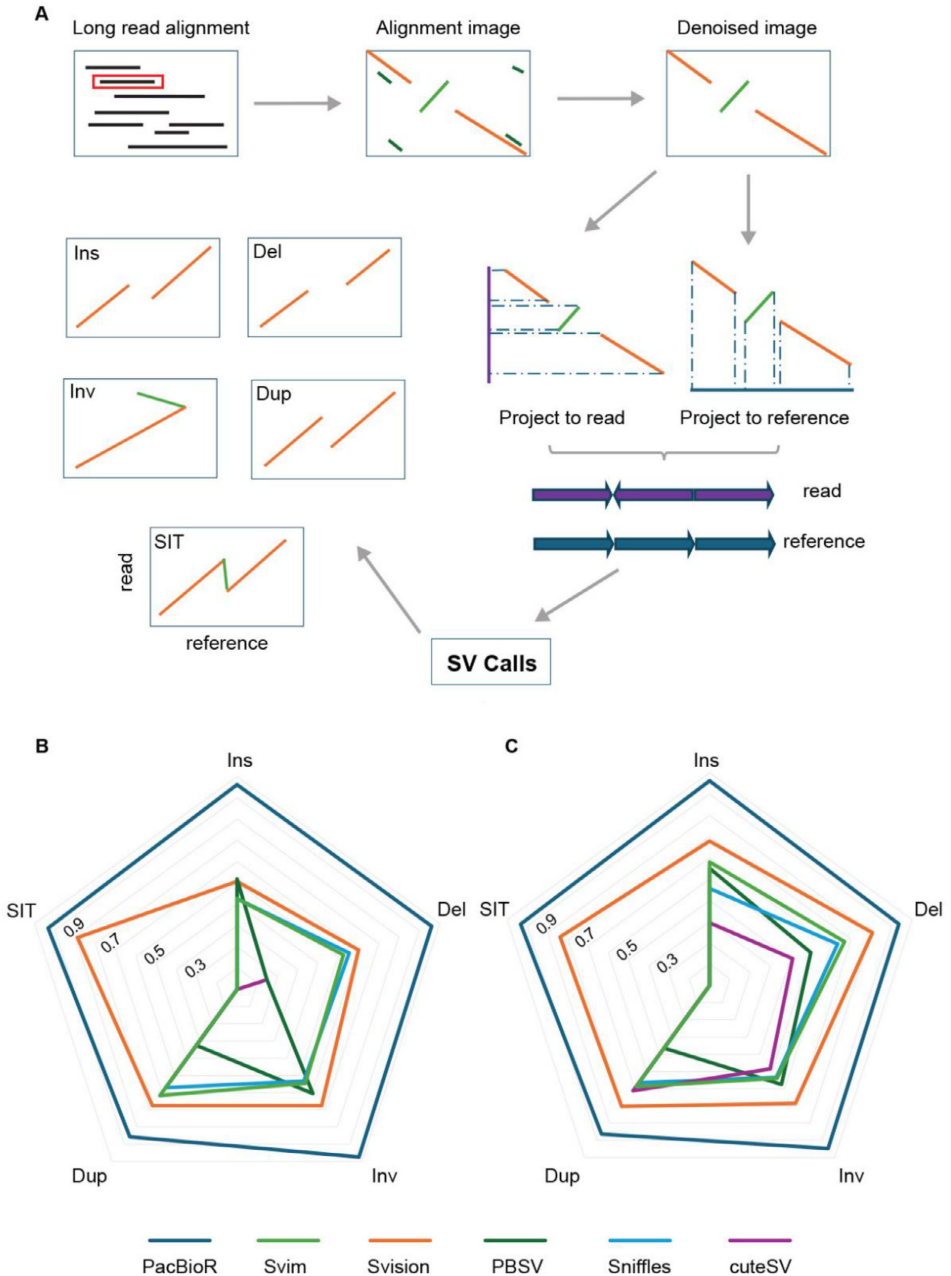
The high‐performance PacBioR compared with different SV callers. (A) Schematic overview of the PacBioR algorithm. The F1 score of simulated SV detection using different tools is shown in (B, C), corresponding to Table S6. HiFi reads were simulated using a modified hg38 genome with different SVs, with 10× genome coverage (B) and 20× genome coverage (C). DEL, INS, INV, DUP, and SIT represent deletion, insertion, inversion, duplication, and small inverted triplication.

Although we confirmed the occurrence of SITs across different cancers, short‐read sequencing has inherent limitations to identify complex SVs, as it often fails to span entire SV regions, which may result in incomplete breakpoint information and inaccurate assembly [43, 44]. To facilitate a more comprehensive investigation of SIT structure, we developed PacBioR, an R package specifically designed to identify various types of SVs, including SITs, from PacBio HiFi reads (Figure 2A). To evaluate the performance of PacBioR, we simulated 2,150 SVs on the hg38 genome using Visor [45] and Sim‐it [46]. These SVs contain 1,000 insertions and deletions, and 50 duplications, inversions, and inverted triplications. For insertion and deletions, we simulated them directly using Sim‐it to generate PacBio HiFi long reads with 10× and 20× genome coverage (Figure 2B,C, Figure S4, and Table S6). For inversions, duplications, and inverted triplications, we simulated 50 events each using Visor and generated 10× and 20× PacBio HiFi reads with the modified hg38 genome using Sim‐it (Figure 2B,C, Figure S4, and Table S6). In addition to PacBioR, we also included other popular SV detection algorithms, including PBSV v.2.11.0 (https://github.com/PacificBiosciences/pbsv), Sniffles v.2.6.3 [47], SVision v1.4 [24], cuteSV v.2.1.2 [20], and SVIM v.2.2.0 [48].

PacBioR achieved high precision and recall for all 5 types of simulate SV events, at both 10× and 20× coverage levels (Figure 2B,C, Figure S3, and Table S6). Almost all tools, except PBSV, could detect deletion events with high accuracy, with SVision having the highest precision and F1 statistics, and PacBioR having the highest recall for deletions. It is interesting to note that all tools except PacBioR suffered low precision for insertions, suggesting insertions are more difficult to detect. SVision is the only other tool that detected inversions with similar accuracy to PacBioR. Moreover, PacBioR detected duplications with much higher accuracy than all other tools, mainly due to its higher recall rate. As SIT is one type of complex SV, besides PacBioR, only SVision could detect it among the tools we evaluated. While SVision has similar precision as PacBioR, it suffers a low recall rate (60% at both 10× and 20× coverage) (Figure 2B,C, Figure S4, and Table S6). Overall, PacBioR outperformed all other tools when detecting insertions, duplications, inversions, and SITs, while displaying similar accuracy to the other tools for deletion events (Figure 2B,C, Figure S4, and Table S6).

### 2.3 Loss of the *rad27* Gene Promotes SIT Formation

To validate the role of FEN1 in the formation of SITs, we investigated its yeast homolog, Rad27, which is highly conserved and functionally equivalent (Figure S11), by performing HiFi sequencing on wild type (WT) and *rad27Δ* yeast cells (Table S7). HiFi sequencing of the WT and *rad27Δ* strains yielded read depths of 919× and 763×, and total reads of 1,102,573 and 1,005,166, respectively (Table 1 and Table S7). Using PacBioR, we identified 742 reads containing SITs in the *rad27Δ* strain, which were clustered into 95 unique events based on their genomic positions, whereas no such events were detected in the WT (Table 1 and Table S8). To validate these results, we performed independent replicate experiments on both strains, which exhibited high reproducibility: WT and *rad27Δ* yielded zero and 95 SITs, respectively (Table 1 and Table S8).

**TABLE 1:** Comparative analysis of SIT events in 17 yeast strains.

Sample	Total reads	SIT reads	SIT events	°C
Wild type	946283	0	0	30
	1102573	0	0	30
	922423	0	0	37
*rad27Δ*	913064	858	95	30
	1005166	742	95	30
	876200	986	230	37
*rad27Δ*_*dun1Δ*	858653	182	35	30
	772996	186	17	37
*rad27Δ_pif1Δ*	969321	3	3	30
*pif1Δ*	917455	4	4	30
*rtt105Δ*	948374	38	38	30
*dna2‐1*	952111	5	5	30
*dun1Δ*	1115480	1	1	30
*exo1Δ*	959880	2	2	30
*mre11Δ*	922525	0	0	30
*mus81Δ*	600256	2	2	30
*rad1Δ*	984584	1	1	30
*sae2Δ*	825403	1	1	30
*sgs1Δ*	999977	0	0	30
*rad51Δ*	918747	0	0	30
*rad27Δ*::RAD27	859210	1	1	30
*rad27Δ*::RAD27‐D179A	979103	44	44	30

**FIGURE 3:**
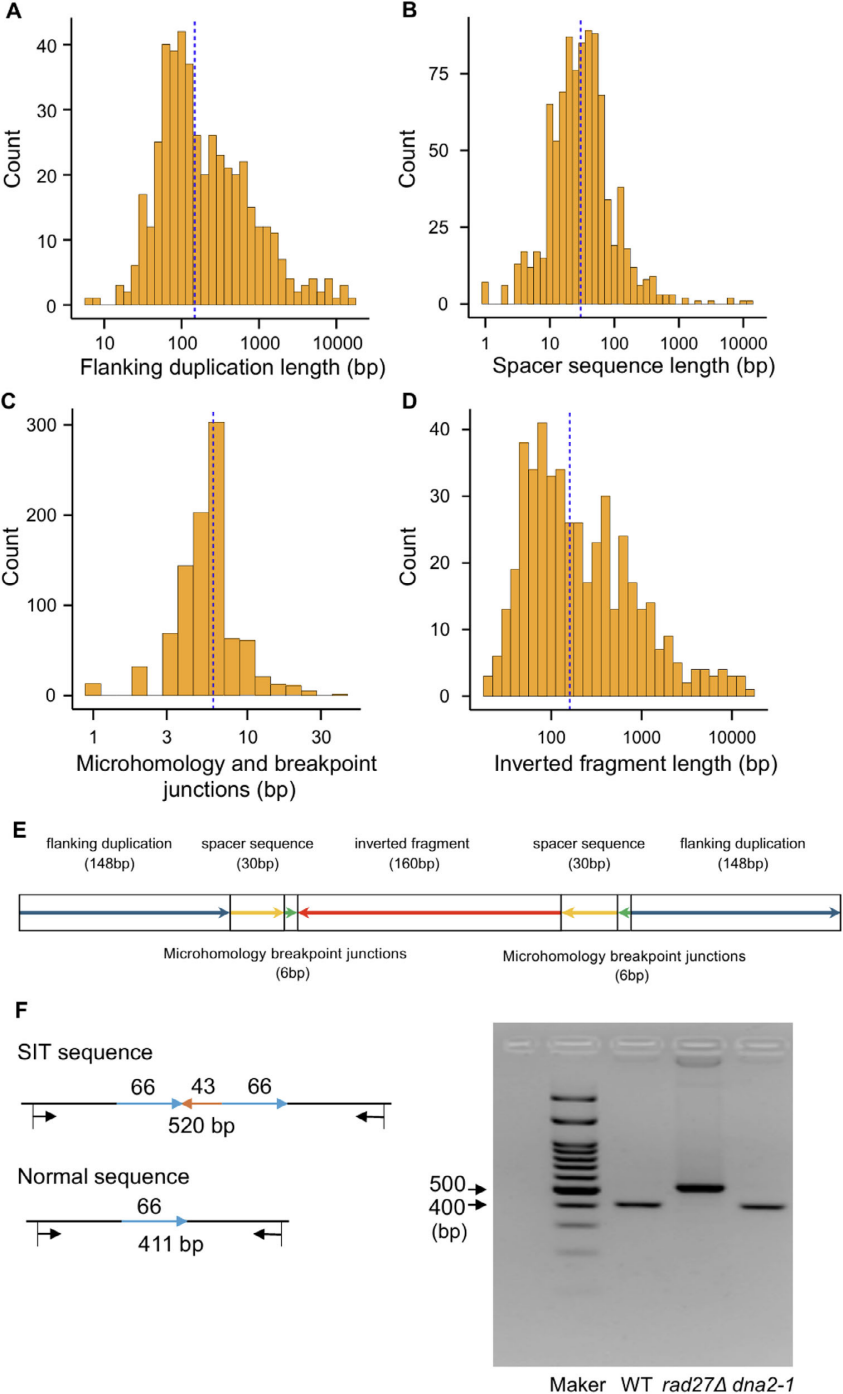
Structure features of SIT events identified in yeast strains. (A) Flanking duplications showed a broad size distribution ranging From 7 bp to > 10 Kbp, With a median of 148 bp. (B) Spacer fragments exhibited a narrow distribution, With most values clustering Around a median of 30 bp. (C) Breakpoint junctions, characterized by reverse complementary sequences, displayed a sharply peaked distribution dominated by 6 bp. (D) Inverted fragments, forming the central reversed fragment, varied substantially in size from 20 bp to several Kbp, with a median length of 160 bp. (E) Schematic representation of a linear SIT structure, illustrating the flanking duplications, spacer sequence, breakpoint junctions, and inverted fragment. (F) PCR validation of a representative SIT event. The expected PCR products of the SIT sequence (520 bp) and the normal locus (411 bp) are shown.

Rad27 is a key nuclease that removes the 5' flap structure during the OFM process [49, 50]. Failure to process the 5' flap may contribute to the formation of SITs. Evidence has been provided that EXO1 and DNA2 also participate in removing 5′ flaps during the OFM process; thus, we generated mutant yeast strains for these two genes (Table S7). We detected two SITs in the *exo1Δ* strain, whereas only five was observed in the *dna2‐1* (Figure 3A and Table S8). Our previous study showed that restrictive temperature (37°C) activates Dun1, which facilitates the transformation of unprocessed 5' flaps into 3' flaps, thereby reinitiating DNA extension [51]. To test this, we did HiFi sequencing analysis on WT, *rad27Δ, dun1Δ*, and *rad27Δ dun1Δ* double knockout strains and detected 0, 230, 1, and 35 SIT events, respectively (Table 1 and Table S8). The number of SIT events in *rad27Δ* mutants at 37°C increased by ≈2.5‐fold compared to the normal condition and decreased ≈6.5‐fold when the *dun1* gene was knocked out (Table 1, Tables S7 and S8). Furthermore, longer 5' flaps are thought to arise by a DNA displacement synthesis mediated by Pol δ and stimulated by Pif1 [52]. We generated *pif1Δ* and *rad27Δ pif1Δ* double knockout strains and the results showed that 4 SIT events in the *pif1Δ* strain and only 3 SIT events in the *rad27Δ pif1Δ* double mutant, respectively (Table 1).

To further validate the specificity of *rad27* phenotype and to test whether Rad27 suppresses SIT formation through its flap endonuclease activity, we performed two complementary rescue experiments in independent *rad27* background strains (Figure S5). One strain expressed a full‐length wild‐type Rad27 protein (*rad27Δ*::RAD27), whereas the other expressed a catalytic‐dead Rad27 variant (RAD27‐D179A), a missense mutation affecting a conserved catalytic residue that abolishes Rad27 flap endonuclease activity (*rad27Δ*::RAD27‐D179A) [53]. The results showed that only one SIT event was detected in the *rad27Δ*::RAD27 strain, whereas 44 SIT events were detected in the *rad27Δ*::RAD27‐D179A strain (Table 1).

Other genes in addition to *rad27* were found to be associated with high rates of global chromosomal rearrangement (GCR) in yeast [4]. To identify additional contributors to SIT generation, we tested six genes that are associated with high rates of GCR, including *mre11*, *rad1*, *sae2*, *mus81*, *rad51*, *and sgs1* (Table 1 and Table S8). The results showed that one SIT event was detected in *rad1Δ*, *and sae2Δ*; two events were detected in *mus81Δ*; and zero events in *mre11Δ*, *rad51Δ*, and *sgs1Δ*; 35 events were detected in *rtt105Δ* strain (Table 1 and Table S8). This result suggested that all these genes are minor contributors to the generation of SIT events. We also examined *rtt105* that encodes an RPA chaperone required for proper RPA loading and stability. The result showed that thirty‐five SIT events were detected in the *rtt105Δ* strain (Table 1), indicating a markedly increased frequency of SIT formation. Reduced RPA availability in the *rtt105Δ* likely facilitates microhomology‐mediated annealing of flap‐derived intermediates [54, 55]. Furthermore, we also examined SIT events in 15 human genomes (Table S9), none of which carried *FEN1* mutations, and no SITs were detected.

### 2.4 Genomic Distribution and Sequence Features of SITs

We analyzed the genomic distribution of SITs and found that they were widespread across the yeast genome (Figure 3A and Table S8). The number of SITs showed a positive correlation with chromosome length. In the *rad27Δ* strain, two independent sequencing experiments identified 190 SITs in different loci with a few overlaps. The highest numbers of events occurred on chromosome (Chr) IV (15 and 16 events, respectively) while the fewest on Chr III (1 and 0 events) (Figure S6 and Table S8). The *rad27Δ* strain maintained at 37°C showed the highest frequency on Chr II (26 events), with the lowest observed on Chr I (1 event) (Figure S6 and Table S8). It is interesting to note that in independent sequencing experiments, we obtained different event distribution patterns. We detected 11 recurrent sites across at least two independent experiments, including 8 sites detected twice, 1 site detected three times, and 1 site detected five times (Figure 3A and Table S8). Finally, among the 574 annotated inverted triplications, 407 overlapped with gene bodies, affecting 465 genes in total, including 84 essential genes representing 7.45% of all essential genes (*n* = 1,127) in yeast (Table S8) [56, 57, 58]. Additionally, nine independent SIT events were detected within the ribosomal DNA region, suggesting that repetitive DNA sequence may promote the formation of SIT. Rad27 is particularly important for stability of microsatellite DNA [59]. We also showed that SIT breakpoints are preferentially located near microsatellite regions, with the distance distribution broadly centered around zero (Figure S12).

We next characterized the sequence features of SITs. The results showed that a complete SIT structure typically consists of four components: the flanking duplication, the spacer sequence, the breakpoint junctions, and the inverted fragment (Figure 3A–E). The flanking duplication showed a broad size distribution ranging from 7 bp to over 10 Kbp, with a median of 148 bp (Figure 3A and Table S8). The spacer fragment exhibited a narrow distribution, with most values clustering around a median of 30 bp (Figure 3B and Table S8). The breakpoint junctions are reverse complementary sequences [29] with a sharply peaked distribution, dominated by 6 bp (Figure 3C and Table S8). The inverted fragment, which forms the central reversed DNA fragment, varied substantially in size from 20 bp to several kilobases, with a median length of 160 bp (Figure 3D and Table S8). Based on these data, we have sketched the linear structure of the SIT. As shown in Figure 3E, SITs exhibit a characteristic DUP/IN/DUP architecture, consisting of two flanking direct duplications (median 148 bp), a central inverted fragment (median 160 bp), two spacer sequences (median 30 bp), and microhomology breakpoint junctions with a median length of 6 bp. In addition, we provided an SIT case that can be validated by PCR amplification in Figure 3F.

### 2.5 SIT Breakpoint Junctions Are Mapped to Okazaki Fragment Junction Points

**FIGURE 4:**
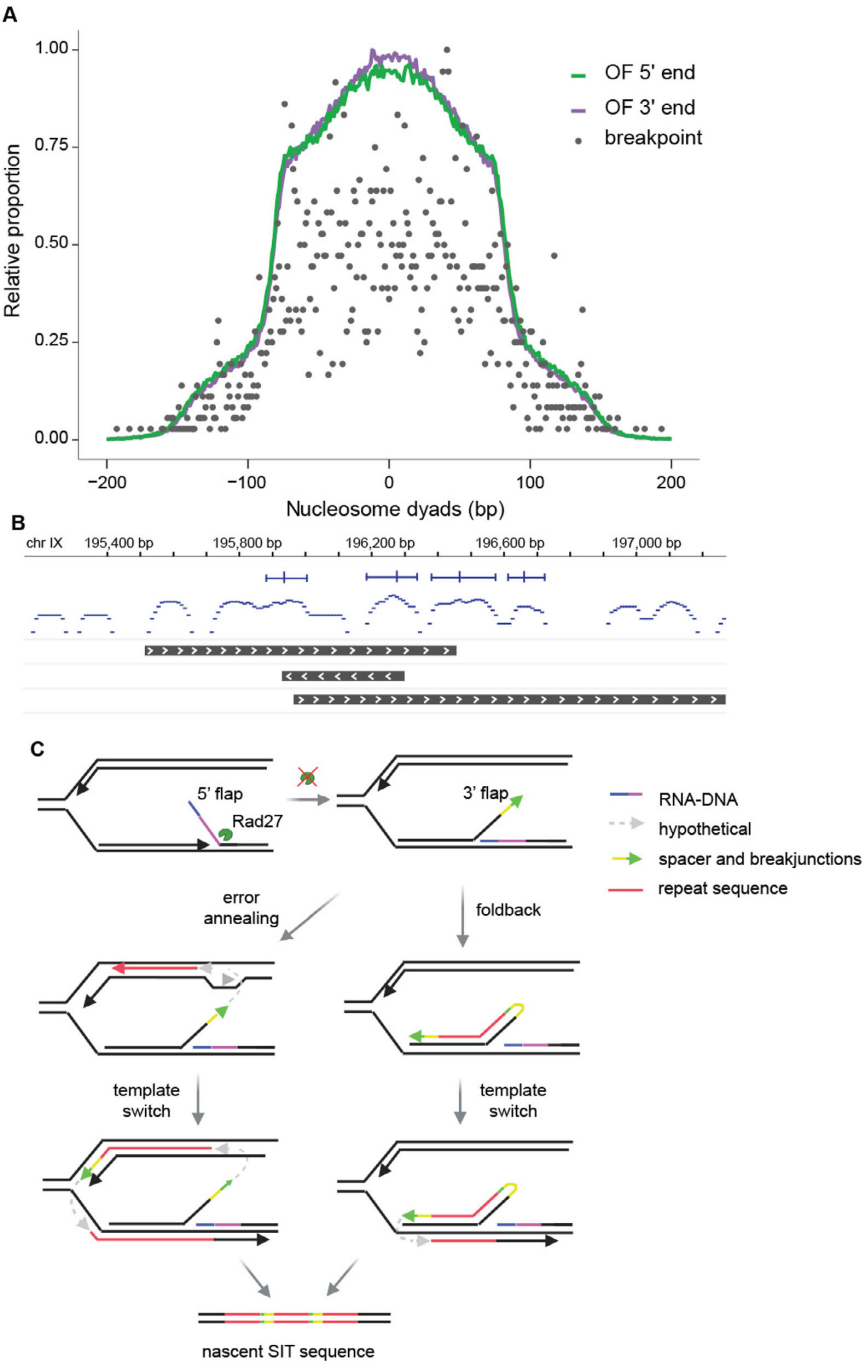
Breakpoint junctions of SITs are associated with nucleosomes and Okazaki fragment junctions. (A) Average distribution of Okazaki fragment 5' ends, 3' ends, and SIT breakpoint junctions relative to nucleosome dyads. (B) Genomic view of a representative SIT structure showing the breakpoint junctions mapped to nucleosome dyads. (C) Modeling SIT formation. When Rad27 is defective and 5' flaps are not removed, 3’ flaps are formed. The 3' flaps may either anneal to complementary sequences or fold back to prime DNA synthesis in the opposite direction, giving rise to an inverted fragment. Subsequent template switching at reverse complementary sequences promotes the nascent fragment to return to the original template and synthesize the DNA fragment one more time, producing a full SIT structure.

The association of major OFM enzyme FEN1 with SIT formation prompted us to investigate whether the SIT breakpoint junctions could be mapped to the Okazaki fragment termini. Previous studies have shown that Okazaki fragment termini preferentially occur near nucleosome midpoints (dyads) in yeast [60]. Therefore, we performed micrococcal nuclease sequencing (MNase‐seq) in both WT and *rad27Δ* strains, and identified 64,624 and 64,631 nucleosomes, respectively (Tables S10– S12). Given that the stereotypical pattern of nucleosome depletion at promoters and well‐ordered nucleosomes in gene bodies is found in all eukaryotes [61, 62, 63, 64, 65], we plotted the average nucleosome profile over all yeast genes (Figure S7). An overlay of hybridization profiles after alignment at the transcription start sites (TSS) showed an apparent nucleosome‐depleted region (NFR) and a regular nucleosome array downstream of the TSS (Figure S7). Nucleosome signals around the transcript termination sites (TTS) were also defined, with a marked drop in coverage at the termination point followed by phased nucleosome occupancy downstream (Figure S7). Meanwhile, 19.81% of nucleosomes exhibited significant changes in position, occupancy, and fuzziness in *rad27Δ* compared to WT (FDR <0.05; Table S13). We further integrated Okazaki fragment sequencing data, and the result showed that the breakpoint junctions of SITs, as well as Okazaki fragment termini, tend to show peak density around dyads (Figure 4A,B and Table S14).

Based on the data presented above, we propose that SITs are generated (Figure 4C) as follows: DNA polymerase δ displacement synthesis displaces the upstream 5’ flap for cleavage by Rad27. When Rad27 is defective, the 5′ flaps are not removed, leading to the formation of 3′ flaps. Formation of flap structures is influenced by factors such as Dun1 and Pif1, which stimulates Pol δ‐driven strand displacement and promotes generation of extended flap intermediates. The 3′ flaps can subsequently invade the sister chromatid and prime DNA synthesis using the nascent DNA strand as a template. When the breakpoint junction sequence is palindromic, the 3’ flap may anneal to the complementary strand and prime DNA synthesis in the opposite direction, or the end sequence may fold back on itself, to form an inverted fragment [66]. This is consistent with our observation that loss of Rad27 function leads to an increase in simple inversions and tandem duplications compared with all other strains analyzed (Figure S10). When the inverted synthesis meets resistance and finds another reverse complement sequence from the template strand of the original chromatin, the DNA polymerase synthesizes the original DNA fragment one more time.

### 2.6 Cellular Instability and Elimination of SIT

**FIGURE 5:**
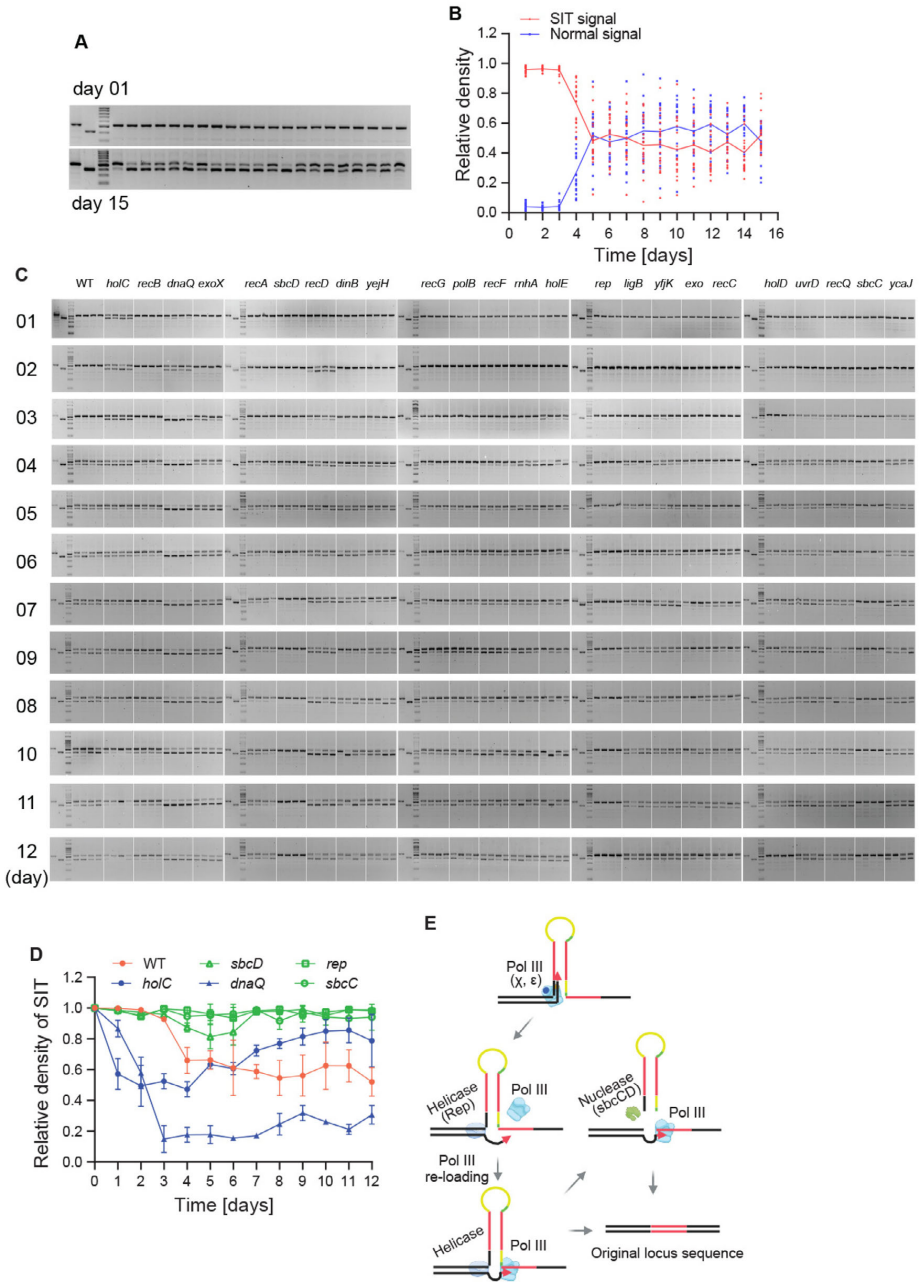
Dynamic equilibrium and regulation of SIT structure in cells. (A) Representative PCR‐based detection of SIT and corresponding normal sequence in WT strain at day 1 and day 15 time points following plasmid transformation. (B) Quantification of SIT (red) and normal (blue) signal intensities in WT strain over time. Signal intensities were normalized to total signal and plotted as relative density. Data represent the mean ± standard deviation calculated from 21 independent clones. (C) PCR‐based monitoring of SIT and normal structure signals over 12 d following plasmid transformation in WT and 24 mutants (Figure S9). (D) Relative density of SIT signal in WT and five representative mutants over time, showing their effects on the SIT structure stability (Figure S9). Error bar represent mean ± standard deviation from three biological replicates. (E) Proposed mechanism depicting how the SIT structures are precisely eliminated by DNA Pol III slippage, helicase displacement of the primer, and structure‐specific nuclease cleavage of hairpin structures.

When the SIT structure was introduced in a plasmid to the *E. coli* and yeast systems, the inverted fragment along with a copy of the duplicated fragment was progressively eliminated and recovered to the original locus sequence (Figure 5A,B and Figure S9). In *E. coli*, the SIT fragment density started to decrease by day 3, reaching ≈50% of the original density by day 4, with the ratio remaining relatively stable thereafter (Figure 5B and Figure S9). Given the rarity and possible genomic impact of SIT, we speculated that organisms may have evolved specific adaptive systems to cope with such rearrangements. We selected 24 genes with known functions in DNA replication, repair, and recombination as candidates to test this hypothesis (Figure 5C, Figure S9, and Table S16). With WT and 24 mutant *E. coli* strains, we show that loss of *holC* and *dnaQ* genes, which encode *E. coli* DNA polymerase III (Pol III) accessory subunits χ and ε, accelerated the SIT elimination processes significantly starting at day 0, even though the SIT band density rebounds beyond the WT density, which is probably due to induced expression of a compensatory gene for *dnaQ* (Figure 5D and Figure S9). Three other genes, including *rep*, which encodes the helicase Rep, and nuclease subunits *sbcC* and *sbcD*, which form the SbcCD structure‐specific nuclease complex, tolerated SIT mutations until Day 15 without popping it out (Figure 5C,D and Figure S9).

Based on this set of data, we propose a model (Figure 5E) on how the SIT structure is eliminated: We first confirmed the formation of hairpin structure or other higher order structures formation using in vitro biochemical assay (Figure S8). *E. coli* DNA Pol III accessory subunits χ and ε enable displacement synthesis of the hairpin structure and replicate the full length of the SIT mutation. Knock out of these two factors leads to hairpin (made of DUP‐IN, spacer, and breakpoint junctions) slippage and accelerated elimination of hairpin structures. Rep helicase separates the synthesized DNA from the hairpin and promotes the slippage process. Meanwhile, the knockout of *sbcC* or *sbcD* inactivates the hairpin‐cleaving activity of the SbcCD endonuclease complex [67], which leads to stabilization of the SIT structure.

## 3 Discussion

SVs are one of the major classes of genetic variation and are closely associated with evolution [68, 69, 70] and human disease [71]. The discovery and characterization of SVs, interpreting their genetic consequences, have been a central focus of genetic research [5]. In this study, we describe a new type of SV in yeast characterized by a DUP/IN/DUP structure, a spacer sequence, and breakpoint junctions, with median lengths of 148/160/148, 30, and 6 bp, respectively. These structural features are highly consistent with those of SITs identified in human cancer genomes (Figure S3). Although they share structural features with locus‐specific inverted triplications, which vary from kilobase to megabase in previously reported cases [40, 41], SITs are clearly distinguished by their substantially smaller size and unique mechanism of formation.

*The observed low frequency of SIT events in human cancer genomes, with 745 samples showing no detectable SITs, may reflect both the inherent rarity and the profound impact that such complex SVs can exert on genome architecture and gene function*. Meanwhile, we cannot exclude the possibility that intrinsic limitations of short‐read sequencing [72] contribute to the low detection frequency of SITs in many cancer samples. While short‐read sequencing technology lacks the resolution to capture many complex SVs, particularly in repetitive or complex genomic regions [73, 74, 75, 76, 77], long‐read PacBio HiFi sequencing offers clear advantages for complex SV detection [75, 78], and our PacBioR software, specifically optimized for this platform, outperforms existing tools in identifying complex SVs, particularly SITs. Thus, integrating PacBio HiFi sequencing with PacBioR may help address these limitations and facilitate genome‐wide analyses of rare and complex SVs.

Among DNA‐associated genes, *FEN1* mutations exhibited the highest OR enrichment, highlighting a close association between FEN1 and SIT formation. This was further validated in yeast, where deletion of the FEN1 yeast homologue *rad27Δ* resulted in a substantial increase in SIT events. Complementary expression of full‐length Rad27 in the *rad27Δ* background efficiently rescued this phenotype and largely eliminated SIT formation, confirming the specificity of Rad27 loss in driving SIT accumulation. The ODIRA model, based on the observation that the inverted triplication contains the origin of replication (*AR*S2*28*), proposes that replication errors at origins, followed by fork reversal and ligation, drive the formation of inverted triplications [27, 41, 42]. *In addition, Carvalho* et al. *proposed that the inverted triplication can arise from the restart of a collapsed replication fork, in which a DNA break generates a 3′ tail that invades the opposite sister strand* [29, 40]. Therefore, we established a predicted model attributing the origination of SIT structure to DNA replication and functional deficiency of the major OFM enzyme, FEN1/Rad27. The unprocessed 5' flap blocks the DNA polymerase δ from displacement synthesis and leads to the formation of a 3' flap, which is capable of sister chromatin strand invasions [50, 51]. When the 3' flap possesses a palindromic sequence, it may invade and anneal with the complementary sequence in the opposite direction. The newly synthesized DNA in the opposite direction must undergo a second template switch, triggered by fork stalling on the original template strand, which has been documented previously [27, 29, 40, 41, 42]. Alternatively, the 3' flap may fold back on itself when the complementary sequence is available [50, 51, 55, 79] and have a template switch at the upstream nick to return DNA synthesis to the original template, generating a SIT structure.

Our study demonstrated the inherent instability and dynamic equilibrium of SIT structures in a cellular context. The functional deficiency of SbcCD suppresses the resolution of inverted triplications, indicating that these rearrangements may form hairpin structures in the cell that are specifically recognized and cleaved by hairpin‐resolving nucleases [67]. Meanwhile, the presence of both an inverted repeat and direct repeat (DR) within an inverted triplication may provide a potential substrate for replication slippage [80, 81, 82]. When replicating a DR, the polymerase may stall and dissociate from the newly synthesized strand, then realign with the second duplication and resume synthesis, causing additions or deletions of repeat units [80, 82]. In vitro studies have shown that the loss of *E. coli* 3' → 5' exonuclease, polymerase II, and the T4 and Φ29 phage polymerases, is correlated with the inhibition of replication slippage [82]. However, our study showed that functional deficiency of DnaQ, which primarily contributes to the 3′→5′ exonuclease activity of DNA Pol III, promoted the resolution of inverted triplications relative to WT. This discrepancy may be attributed to differences in experimental systems or enzymatic contexts. Our results indicated that the deletion of *holC*, which stabilizes DNA polymerase III on single‐stranded DNA via interaction with single‐stranded DNA‐binding protein, accelerated the repair of inverted triplications. This finding is consistent with previous studies showing that single‐stranded DNA‐binding proteins can suppress the occurrence of replication slippage. In addition, we found that slippage is likely promoted by the helicase activity of Rep. When the polymerase stalls, Rep may bind to the end of the nascent strand end and initiate unwinding along the DNA in the 3′ to 5′ direction [83, 84, 85], thereby facilitating strand dissociation during the slippage process.

In summary, the SIT structure as a novel SV has been characterized in this study. The PacBioR package offers a robust and scalable method for detecting complex SVs, particularly SIT structures. Our mechanistic analyses demonstrate that replication errors during OFM are a major driver of SIT formation and provide two possible pathways underlying its origin. We further propose two repair routes for the SIT structure, replication slippage and hairpin cleavage, which together help explain the inherent instability and dynamic maintenance of SIT structures in vivo. The rarity of SITs likely reflects both the stringent conditions required for their formation and evolved cellular repair systems, whereas imperfect repair may account for the frequent co‐occurrence of other SVs at the same loci [29, 40, 42]. This study also has limitations, as our mechanistic dissection was confined to yeast and *E. coli*, and its applicability to mammalian systems remains to be established. In addition, the molecular events driving the second template switch during SIT formation remain unresolved. Addressing these gaps is essential for the refinement of our model and further clarifying the role of SITs in genome stability and tumorigenesis.

## 4 Experimental Methods

### 4.1 Cancer Genome Datasets Collection and Analysis

We collected 1,340 short‐read sequencing datasets, including both 739 whole‐genome sequencing (WGS) and 591 whole‐exome sequencing (WES) samples, derived from 29 projects covering 22 distinct cancer types (Figure 1A and Table S1), for use in this study. All short‐read sequencing datasets used in this study were obtained from the NCBI Sequence Read Archive (https://www.ncbi.nlm.nih.gov/sra/), except for the data from B‐ALL patients, which were generated by our laboratory (Table S1). Paired‐end reads containing adapters, ploy‐N, or any bases with Phred quality scores lower than 20 were removed with Cutadapt v.2.1 and TrimGalore v.0.6.5 (https://www.bioinformatics.babraham.ac.uk/projects/trim_galore/). The clean reads were aligned to the hg38 reference genome using Bowtie2 v.2.2.9 [86]. Duplicated reads were removed by the Picard v.2.23.4 MarkDuplicates module (https://broadinstitute.github.io/picard/index.html). The Pindel v.0.2.5 [12] was used to call SVs from the alignment data. We filtered the Pindel results by requiring the length of the alternative sequence (ALT) to be more than twice that of the reference sequence (REF) in one SV. Then the alternative sequences were re‐aligned to the reference genome using Blastn v.2.16.0 [87], and candidate SITs were filtered by identifying alignment patterns featuring a central reverse‐oriented segment flanked by two direct fragments. The SITs were subsequently confirmed through manual inspection using Integrative Genomics Viewer (IGV) (Figure S1A) [88]. The SNVs and indels were identified by VarScan2 v.2.3.7 [89]. All variants were annotated using Annovar (http://www.openbioinformatics.org/annovar/) [90].

### 4.2 Yeast Strains and PacBio HiFi Sequencing

The WT yeast used in the study was the haploid MATa RDKY2669 strain (Table S7). Single gene deletion derivatives of WT, including *rad27*, *rad1*, *rad51*, *dun1*, *mre11*, *mus81*, *sgs1*, *sae2*, were generated via a homologous recombination‐based strategy. WT cells were first transformed with PCR‐amplified DNA fragments containing *his3*, *trp1*, or *ura3* as selection markers, flanked by 39 bp homologous arms corresponding to the target loci (Tables S7 and S16). Stable transformants were then selected using corresponding auxotrophic plates, and successful gene knockout was confirmed by Sanger DNA sequencing. The *dna2‐1* and *exo1* mutant strains were gifted by Judith L. Campbell (California Institute of Technology).

Unless otherwise specified, all yeast strains were cultured in YPD medium at 30°C with shaking at 200 rpm. Yeast cells were inoculated at an initial OD600 of 0.1 into liquid YPD medium and grown overnight, and then DNA was extracted using the YeaStar Genomic DNA Kit (ZYMO Research, D2002) according to the manufacturer's instructions. After confirming the quality of the genomic DNA, the SMRTbell libraries were prepared with SMRTbell Prep Kit 3.0 (Pacific Biosciences) following the manufacturer's protocol. Briefly, The Megarupter 3 system (Diagenode, Denville, NJ, USA) was used for genomic DNA shearing, and the Blue Pippin system (Sage Science, Beverly, MA, USA) was used for size‐selection to keep fragments greater than 13 Kbp. A subset of the libraries was constructed using barcoded adapters to allow pooling before size‐selection. Each library was run on one SMRT Cell (version 8 M) using version 2.0 sequencing reagents. The Sequel IIe sequencer (Pacific Biosciences, Menlo Park, CA, USA) instrument was used to generate the raw subreads. The circular consensus sequences were extracted from the subreads and then filtered for short (<100 bp) or low quality (QV <80) using the SMRTLink v. 9.0.0.92188 package to produce the HiFi reads. Samples treated at 37°C were initially cultured at 30°C, then incubated at 37°C for 4 hours, with all subsequent procedures conducted in parallel with untreated samples.

For Rad27 rescue experiments, the Rad27 coding sequence lacking the stop codon was amplified from WT yeast genomic DNA using primers Rad27‐F1 and Rad27‐R1 (Table S16). The PCR product was cloned in frame with a C‐terminal 5×FLAG tag into the pFA6a‐5×Flag‐hphMX6 vector using the In‐Fusion Snap Assembly kit (Takara, Cat. No. 638948) according to the manufacturer's instructions. A nuclease‐deficient mutant (Rad27‐D179A) was generated by overlap‐extension PCR using mutagenic primers D179A‐F and D179A‐R (Table S16) and cloned in parallel. After sequence verification, the resulting RAD27‐5×FLAG‐ADH1 terminator cassette (WT or D179A) was amplified using primers Rad27‐F2 and Rad27‐R2 (Table S16) and subcloned downstream of the ADH1 promoter in the yeast expression vector pRSII424. The constructs were introduced into the *rad27Δ* strain using the lithium acetate transformation method, and transformants were selected on tryptophan dropout medium. Expression of Rad27‐5×FLAG proteins was confirmed by Western blotting prior to subsequent analyses.

### 4.3 Development of PacBioR for Detecting SVs Using HiFi Reads

To improve the sensitivity of inverted triplication detection, we have implemented an algorithm called “PacBioR” using an accurate alignment and rule‐based SV calling scheme (Figure 2). The reads were first aligned to a reference genome using pbmm2 (https://github.com/PacificBiosciences/pbmm2). The alignments were then examined for long stretches of insertion, deletion, or soft clips. Reads with these abnormalities were considered candidates for further analysis. The MegaBLAST algorithm [91] was used to realign the candidates with improved accuracy. To overcome its slow speed, we utilized a high‐speed version, hs‐blastn [92], which is more than 20× faster than regular megaBLAST. Several optimization steps were implemented to resolve the complex alignment structure. These steps included: resolving multiple alignment regions among distal regions, removing redundant alignments within a local region, and filtering out the alignments with a large number of mismatches. These cleaned alignment results were then converted into two vectors, one representing the alignment along the query read and the other representing the alignment along the reference genome. This can be achieved by projecting the aligned segments onto query and reference sequences, with 1 representing a match, 0 representing a missing base, and ‐1 representing a match on the opposite strand. The two vectors serve as the foundation for identifying candidate SVs. For example, if the query vector contains a stretch of 0 and the reference vector contains no 0, the read contains an insertion. Deletion events will have the opposite pattern. Duplications are represented by a stretch of integers greater than or equal to 2 in the query vector, while the reference vector contains only 1. Inversions have a stretch of ‐1 in the query. Inverted duplications can be indicated by both 1 and ‐1 stretches in the query and a stretch of integers larger than 2 in the reference. This detection process was repeated in a sliding window approach to cover all segments in each query read, ensuring multiple SV events on each read could be detected as well. Each read was examined independently and assigned an SV event based on the processes outlined above. Similar SV events were then aggregated, and the number of supporting reads was recorded along with the total coverage at these loci.

### 4.4 MNase‐Seq and Data Analysis

The mono‐nucleosomes were extracted from WT or *rad27Δ* yeast cells (Table S9) according to a previously described protocol [93] with a small modification. Cells were grown in YPD medium in stable log phase (OD600 ≈ 0.6) and harvested by centrifugation. The cells were fixed with formaldehyde at a final concentration of 1% for 15 min, followed by quenching with glycine at a final concentration of 125 mm for 5 min. The cells were then pelleted and washed twice with cold sterile water. The pellet was resuspended with lysis buffer (1 m sorbitol, 50 mm Tris–HCl, pH 7.4, 10 mm *ß*‐mercaptoethanol), and zymolyase solution (10 mg mL^−1^ zymolyase dissolved in 40% glycerol, 60% lysis buffer) was added at a final concentration of 0.25 mg mL^−1^. Cells were incubated at 30°C with gentle shaking, and then the spheroplasts were collected by centrifugation at 3000 *g* for 10 min at 4°C. The spheroplasts were gently resuspended in resuspension buffer (0.5 mm spermidine, 0.075% NP‐40, 50 mm NaCl, 10 mm Tris–Cl, pH 7.5, 5 mm MgCl_2_, 1 mm CaCl_2_, 1 mm β‐mercaptoethanol) and digested with micrococcal nuclease (300 U µL^−1^) at 37°C for 30 min. The reaction was stopped on ice by adding EDTA to a final concentration of 500 mm. DNA was purified using phenol–chloroform extraction and concentrated by ethanol precipitation. The integrity and size distribution of the digested fragments were determined using the microfluidics‐based platform Bioanalyzer (Agilent). A PCR‐free protocol with the KAPA Library Preparation kit (Roche) was employed to prepare short‐insert paired‐end libraries for MNase sequencing. The libraries were sequenced using TruSeq SBS Kit v4‐HS (Illumina) following the manufacturer's protocol. The raw reads were quality checked by FastQC v.0.20.0 (https://www.bioinformatics.babraham.ac.uk/projects/fastqc/). The reads containing adapters, ploy‐N, or any bases with Phred quality scores lower than 20 were removed with Cutadapt v.2.1 and TrimGalore v.0.6.5 to generate clean reads. The reads were then aligned to the reference yeast genome sacCer3 using Bowtie2 v2.2.9. Nucleosome positioning and occupancy were examined by DANPOS2 v.2.2.2 [94] using the function Dpos.

### 4.5 Human HiFi Sequencing Data Collection

Leukemic cells from the bone marrow aspirates of three patients diagnosed with B‐ALL were collected at diagnosis and isolated by density gradient centrifugation. Samples of <70% tumor cell content were purified by fluorescence‐activated cell sorting. Genomic DNA was extracted from the cells using a commercial DNA extraction kit (PureLink Genomic DNA Mini Kit, Invitrogen, Carlsbad, CA, USA). After quality check of the genomic DNA, HiFi library preparation and sequencing were performed following the same protocol used for yeast samples. In addition, we incorporated 12 publicly available HiFi sequencing datasets into our study, including data derived from breast carcinoma, melanoma, peripheral blood, lymphoblastoid cells, and the Human Genome Structural Variation Consortium (Table S9).

### 4.6 *E. coli*‐Based Functional Screening of Factors Involved in Inverted Triplication Stability

PCR amplification of normal and SIT fragments was performed using genomic DNA, 12.5 µL of 2× Taq PCR Premix (Bioland Scientific), and 0.2 µm of forward and reverse primers (Table S15). PCR conditions were 95°C for 5 min followed by 35 cycles of 95°C, 5 sec; 58°C, 30 sec; 72°C, 45 sec; with a final elongation step of 72°C for 10 min. The PCR‐amplified inverted fragments were ligated into TOPO TA cloning vectors and subsequently transformed into *E. coli* wild‐type and 23 single‐gene knockout strains (Horizon, catalog#OEC4987) (Table S14). Transformed cells were plated on LB agar supplemented with ampicillin (100 µg mL^−1^) and grown overnight at 37°C. Three independent single colonies were picked for each strain and cultured into 4 mL of LB liquid medium supplemented with ampicillin (37°C, 270 rpm). After 24 h, 4 µL of the culture was transferred into fresh 4 mL LB medium under the same conditions. After 24 hours of growth, 4 µL of the bacterial culture was transferred into 4 mL of fresh medium. This process was repeated under the same conditions for sequential subcultures. At each passage, PCR analysis was conducted to evaluate the status of both the normal and inverted triplication fragments. The densities of the SIT and normal fragments were quantified using ImageJ software from PCR gel images.

### 4.7 Nuclease S1 Foot Printing Assay

To test whether SIT sequences can form hairpin‐like higher‐order structures, representative SIT sequences were used to design oligonucleotides (Table S16). The oligonucleotides contained the essential structural elements required for hairpin formation, including the inverted repeat and the loop region. The 5′ end of the oligonucleotide was labeled with 6‐carboxyfluorescein (FAM) for fluorescence detection. For structure formation, oligonucleotides were dissolved in annealing buffer (10 mm Tris‐HCl, 1 mm EDTA), heated to 95°C for 5 min, incubated at 72°C for 10 min, and then gradually cooled to room temperature. Annealed oligonucleotides were stored at 4°C until use. For S1 nuclease digestion, annealed oligonucleotides were incubated with the indicated concentrations of S1 nuclease in the corresponding reaction buffer at room temperature. Reactions were terminated by addition of 0.5 m EDTA followed by incubation at 70°C for 10 min. Digestion products were then analyzed to evaluate the presence of single‐stranded regions associated with higher‐order DNA structure formation.

## Funding

This work was supported by funding from the National Cancer Institute (NCI)/National Institutes of Health (NIH) [R01 CA073764 and R01 CA279840 to B.H.S., R50 CA211397 to L.Z.]; and CCSG [P30 CA033572] to City of Hope.

## Conflicts of Interest

The authors declare no conflicts of interest.

## Supporting information




**Supporting File 1**: advs75211‐sup‐0001‐SuppMat.docx.

**Supporting File 2**: advs75211‐sup‐0002‐TableS1‐S16.xlsx.

## Data Availability

Sequence data have been deposited at NCBI as Submission ID PRJNA1348743 (Reviewer link: https://dataview.ncbi.nlm.nih.gov/object/PRJNA1348743?reviewer = 6f3m2gv8k4ojjtcu61jkllhvup) and are publicly available as of the date of publication. Original code for PacBioR has been deposited at GitHub and is publicly available at https://github.com/xiweiwu/pacbior as of the date of publication. All other data reported in this paper will be shared by the corresponding authors upon request.
