# Supplementary material for: DNA Replication Errors Drive Genome‐Wide Small Inverted Triplication Dynamics
Source: Adv Sci (Weinh). 2026 Apr 7:e21949. Online ahead of print. doi: 10.1002/advs.202521949 (PMC13334657; doi:10.1002/advs.202521949)
Supplement: Supplementary file 1 — Supporting File 1: advs75211‐sup‐0001‐SuppMat.docx. [file ADVS-9999-e21949-s001.docx]

Supporting Information

**DNA replication errors drive genome-wide small inverted triplication dynamics**

*Yi Lei^1#^, Yu Zhou^1#^, Haitao Sun^1,3^, Hang Yuan^1^, Xinyu Pei^1^, Jessica D. Hess^1^, Yao Yan^1^, Zunsong Hu^2^, Mian Zhou^1^, Zhaohui Gu^2^, Li Zheng^1***^, Xiwei Wu^2**^, Binghui Shen^1*^*

^1^Department of Cancer Genetics and Epigenetics, Beckman Research Institute, City of Hope, 1500 East Duarte Road, Duarte, California, CA91010, USA

^2^Department of Computational and Quantitative Medicine, Beckman Research Institute, City of Hope, 1500 East Duarte Road, Duarte, California, CA91010, USA

^3^Current addresses: Institute of Medicinal Plant Development, Chinese Academy of Medical Sciences & Peking Union Medical College, Beijing, China

^#^These authors contributed equally

*Correspondence: [bshen@coh.org](mailto:bshen@coh.org)

**Correspondence: [xwu@coh.org](mailto:xwu@coh.org)

***Correspondence: [lzheng@coh.org](mailto:lzheng@coh.org)





**Figure S1. Workflow for the detection and validation of SIT events using short-read sequencing data.**

**(A)** Pipeline for SIT event identification from short-read sequencing data. SVs were initially called using Pindel and filtered to retain candidate events by ≥ 3 supporting reads and with an alternative/reference length ratio > 2. Candidate events were extracted, split-aligned to the reference genome using Blastn, and further filtered to identify SIT structures. Putative SIT events were validated through IGV visualization to confirm the characteristic structure, defined by a central inverted segment flanked by two direct copies. **(B)** Template-switch alignment of SIT structure. The top panel shows a template-switch alignment plot, in which red segments represent forward alignments and green segments represent reverse alignments. The bottom panels display separate alignments: the forward alignment (left, red) corresponding to the inverted segment, and the reverse alignment (right, green) corresponding to the direct copies. **(C)** Representative IGV visualization of a SIT event. Short reads are shown aligned to the reference genome. The SIT configuration is defined by a central inverted segment flanked by two direct copies, as indicated by the schematic alignment track at the bottom.





**Figure S2. GO biological process enrichment analysis of genes associated with high SIT incidence group.**

Bubble plot showing the top 20 significantly enriched GO biological processes among genes significantly enriched in the high SIT incident group. The analysis reveals that genes involved in nucleic acid metabolism and genome maintenance, including RNA splicing, DNA replication, recombination, and repair. Detailed enrichment results are provided in Table S4.


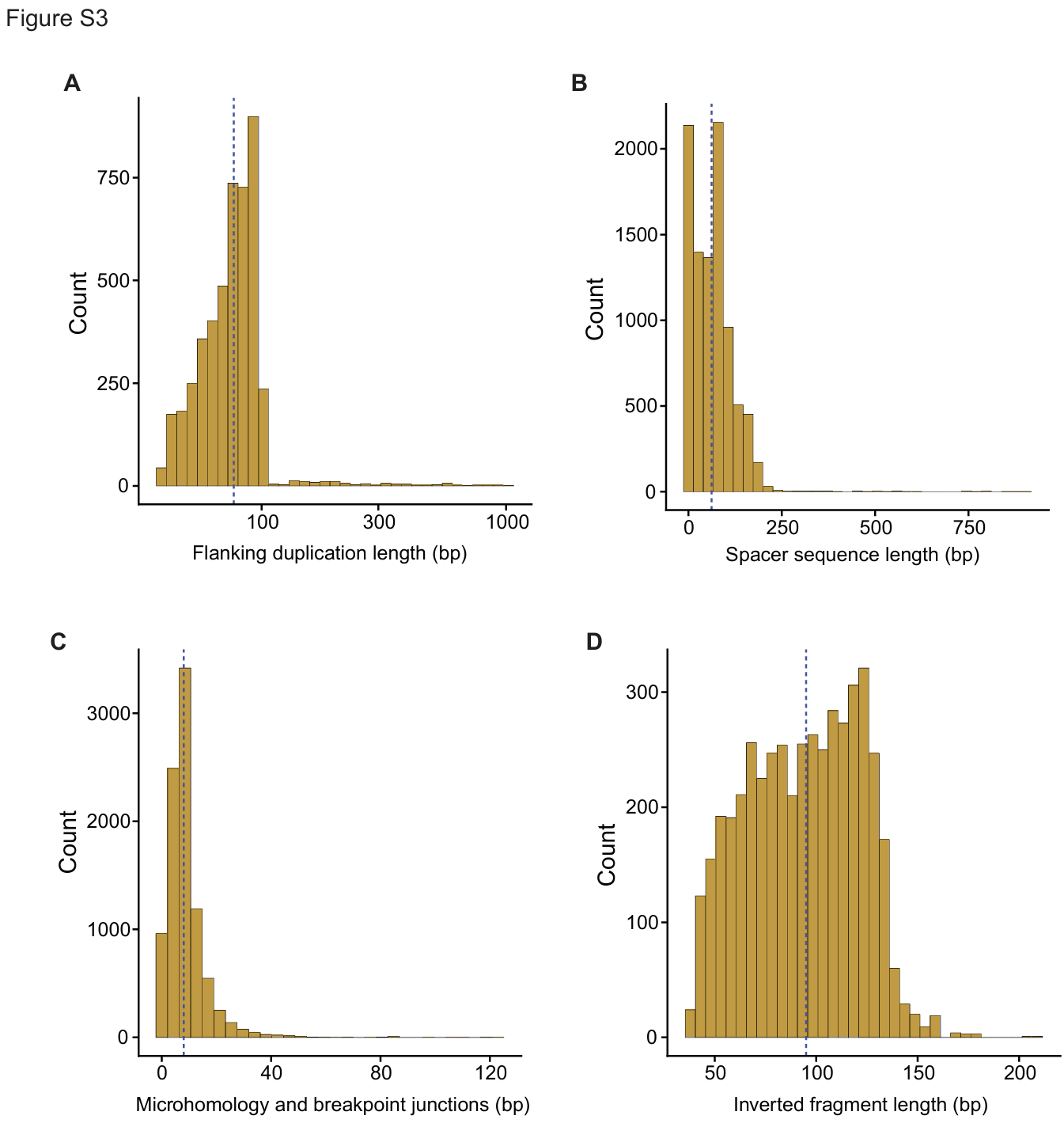


**Figure S3. Structure features of SIT events identified from cancer samples.**

**(A)** Flanking duplication length. The lengths of flanking duplications associated with SIT events show a broad distribution, ranging from a few base pairs to over 1 kb, with a median length of 77 bp. **(B)** Spacer sequence length. Spacer fragments separating the duplicated and inverted segments display a relatively narrow size distribution, with a median of approximately 62 bp. **(C)** Microhomology at breakpoint junctions. Breakpoint junctions are characterized by short reverse-complementary microhomologies. The distribution is sharply peaked, with a median of approximately 8 bp. **(D)** Inverted fragment length. The central inverted fragments vary substantially in size, spanning from tens to several hundred base pairs, with a median length of 95 bp.


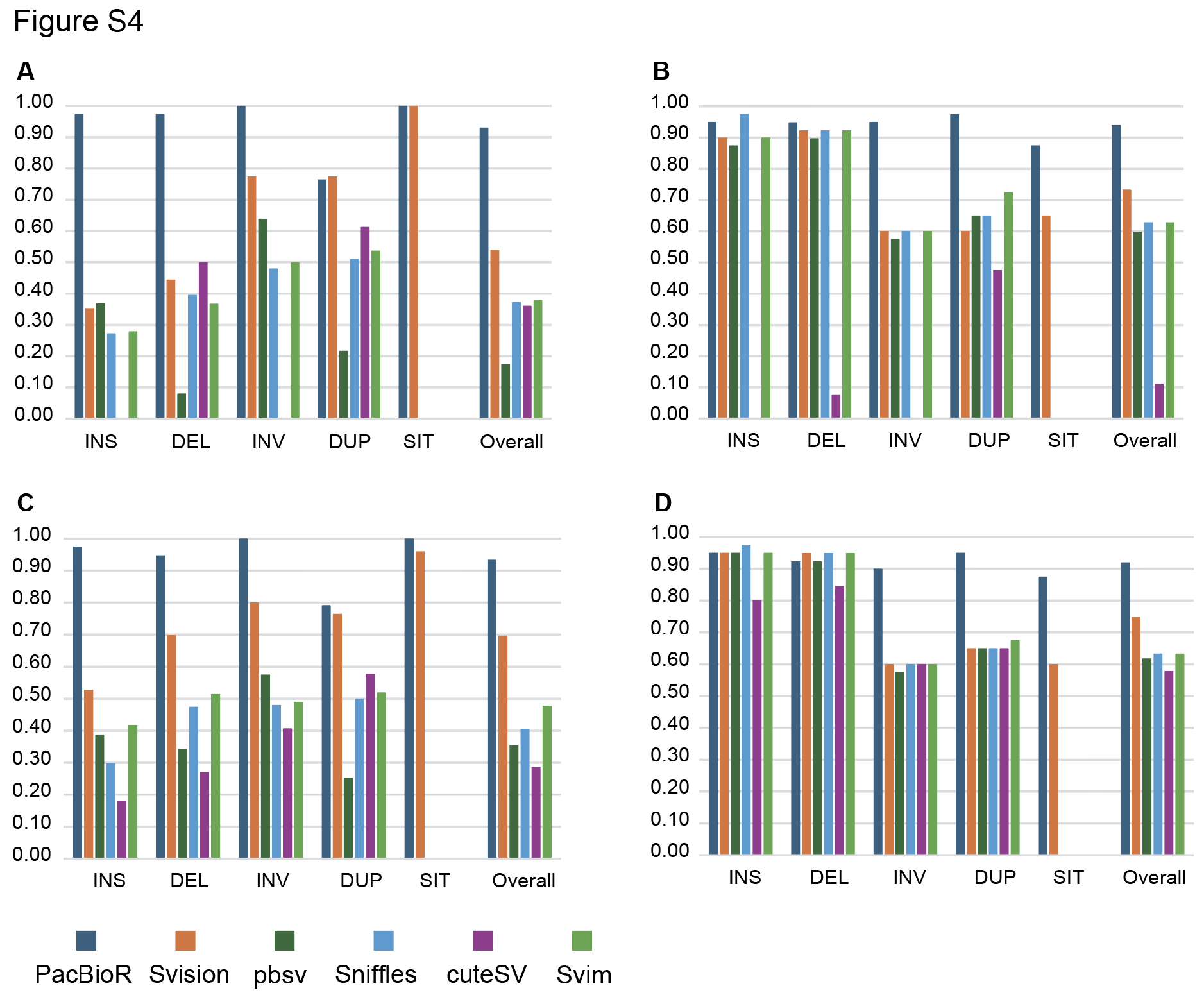


**Figure S4. Comparison of SV caller performance across different sequencing coverage depths.**

Performance of six SV callers (PacBioR, Svision, pbsv, Sniffles, cuteSV, and svim) was evaluated at two sequencing coverage depths (10× and 20×). Precision **(A)** and recall **(B)** of each SV caller at 10× coverage. Precision **(C)** and recall **(D)** of each SV caller at 20× coverage. Performance was assessed across different SV types, including insertions (INS), deletions (DEL), inversions (INV), duplications (DUP), small inverted triplication (SIT), as well as overall performance across all SV categories.


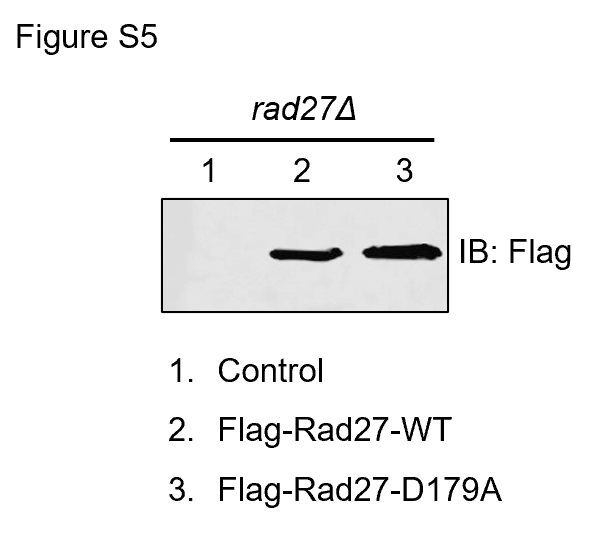


**Figure S5. Validation of Rad27 rescue strain by Western blot.**
Whole-cell extracts from the *rad27Δ* strain and *rad27Δ* strain expressing FLAG-tagged wild-

type Rad27 (FLAG-Rad27-WT) or the nuclease-deficient mutant Rad27-D179A were analyzed

by Western blot using an anti-FLAG antibody. Lane 1, control (*rad27Δ* strain); lane 2,

*rad27Δ::*FLAG-RAD27; lane 3, *rad27Δ::*FLAG-RAD27-D179A. FLAG-Rad27-WT and FLAG-

Rad27-D179A were expressed at comparable levels, confirming successful expression of the

rescue constructs.


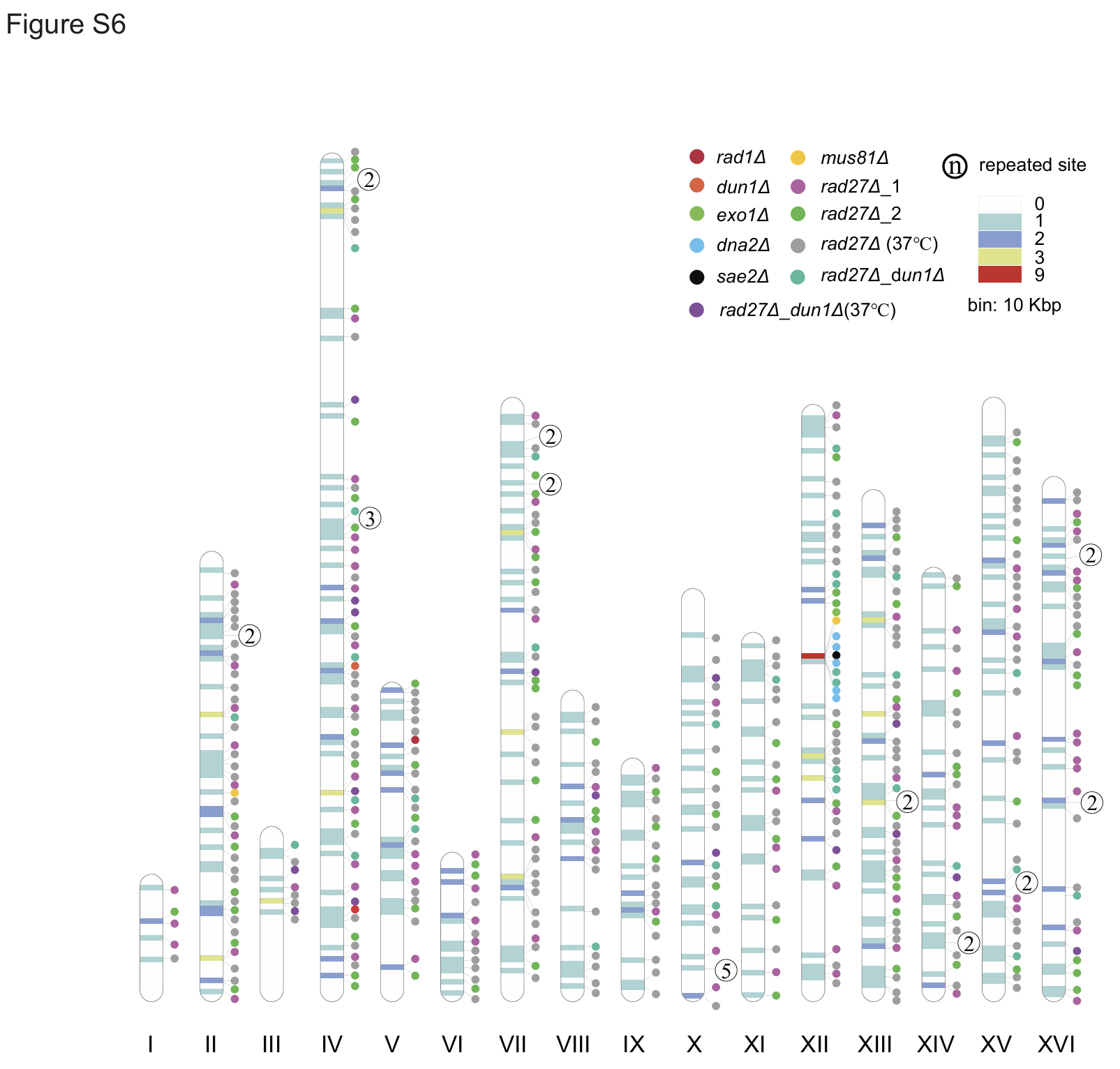


**Figure S6.** **Genome-wide distribution of SIT events in yeast strains.**

Each dot represents a SIT event, color-coded by yeast strain and experimental conditions. "repeated site" indicates how many of the 17 sequencing datasets detected the SIT event at the same locus.


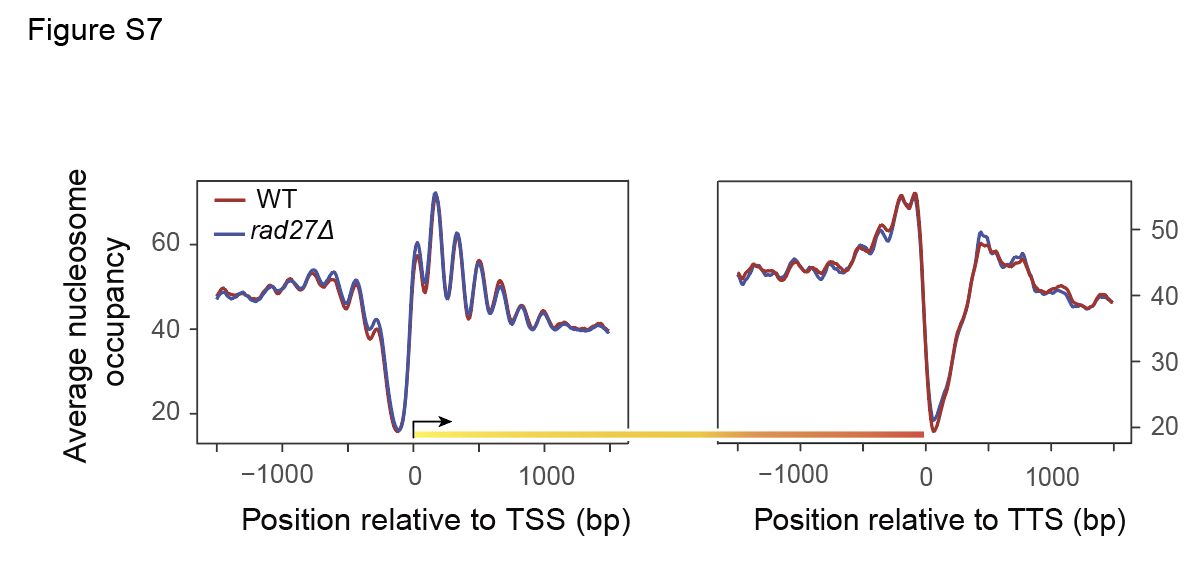


**Figure S7. Nucleosome occupancy profiles aligned at transcription start sites (TSS) and transcription termination sites (TTS).**

The left panel shows a nucleosome-depleted region (NDR) at the TSS, followed by a regular phased nucleosome array downstream. The right panel shows nucleosome occupancy around the TTS, with a sharp drop in coverage at the termination point and phased nucleosome positioning downstream.


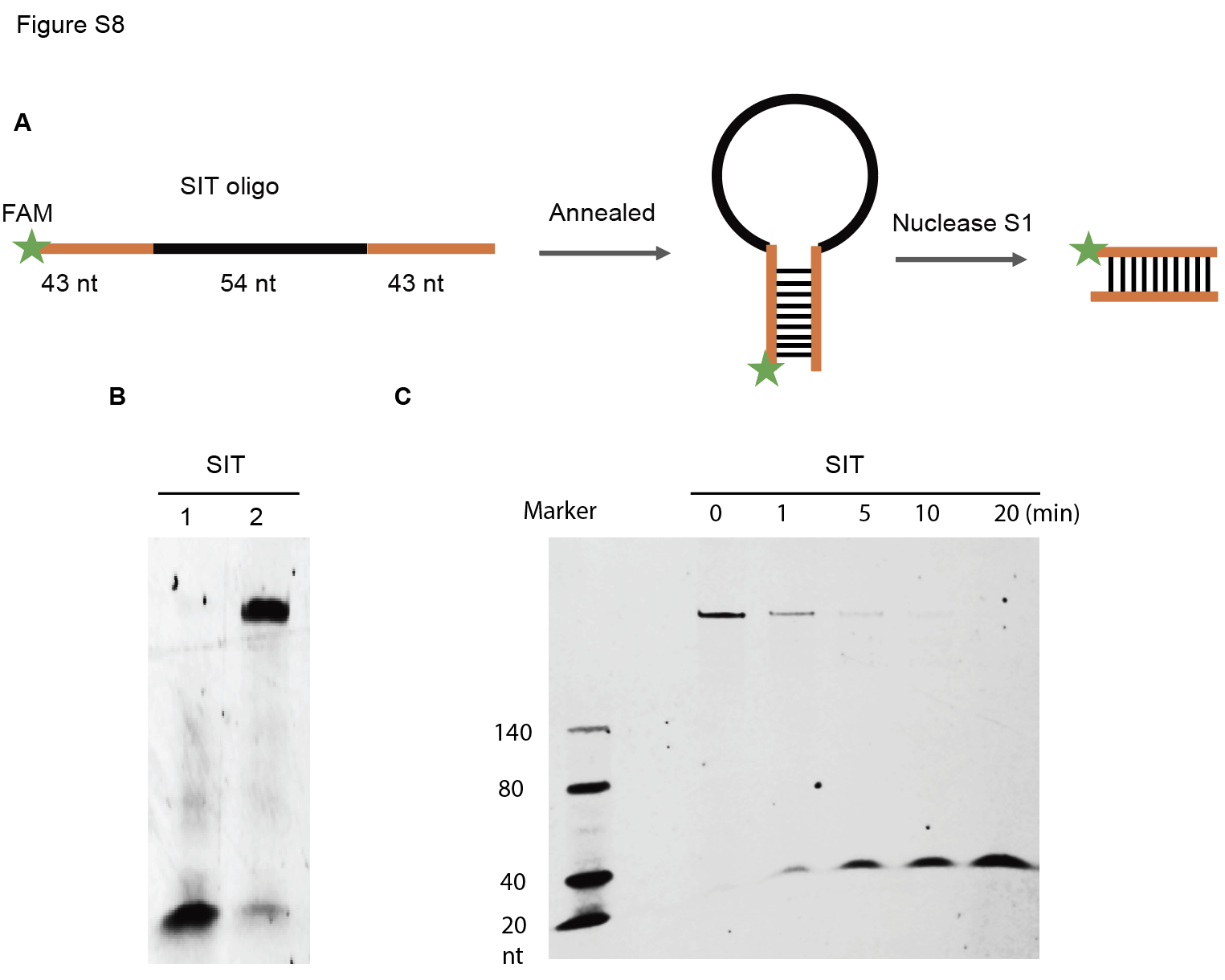


**Figure S8. *In vitro* biochemical validation of higher order structure formation from SIT sequence. (A)** Schematic of the SIT-derived oligo used for biochemical assays. The oligo consists of two 43-nt inverted repeats separated by a 54-nt loop region and is labeled with FAM at the 5′ end. Upon annealing, the SIT oligo forms a fold-back hairpin–like structure that is sensitive to single-strand–specific nuclease digestion. **(B)** 6% native PAGE analysis of annealed substrates containing a representation SIT structure. lane 1 indicates the single-stranded oligo (100 nM); lane 2 indicates the annealed oligo (100 nM). **(C)** Nuclease S1 foootprinting assay of annealed SIT-containing oligo. The substrate was incubated with 10 U of nuclease S1 for the indicated times (0, 1, 5, 10, and 20 min) at room temperature, followed by 12% denature PAGE analysis.


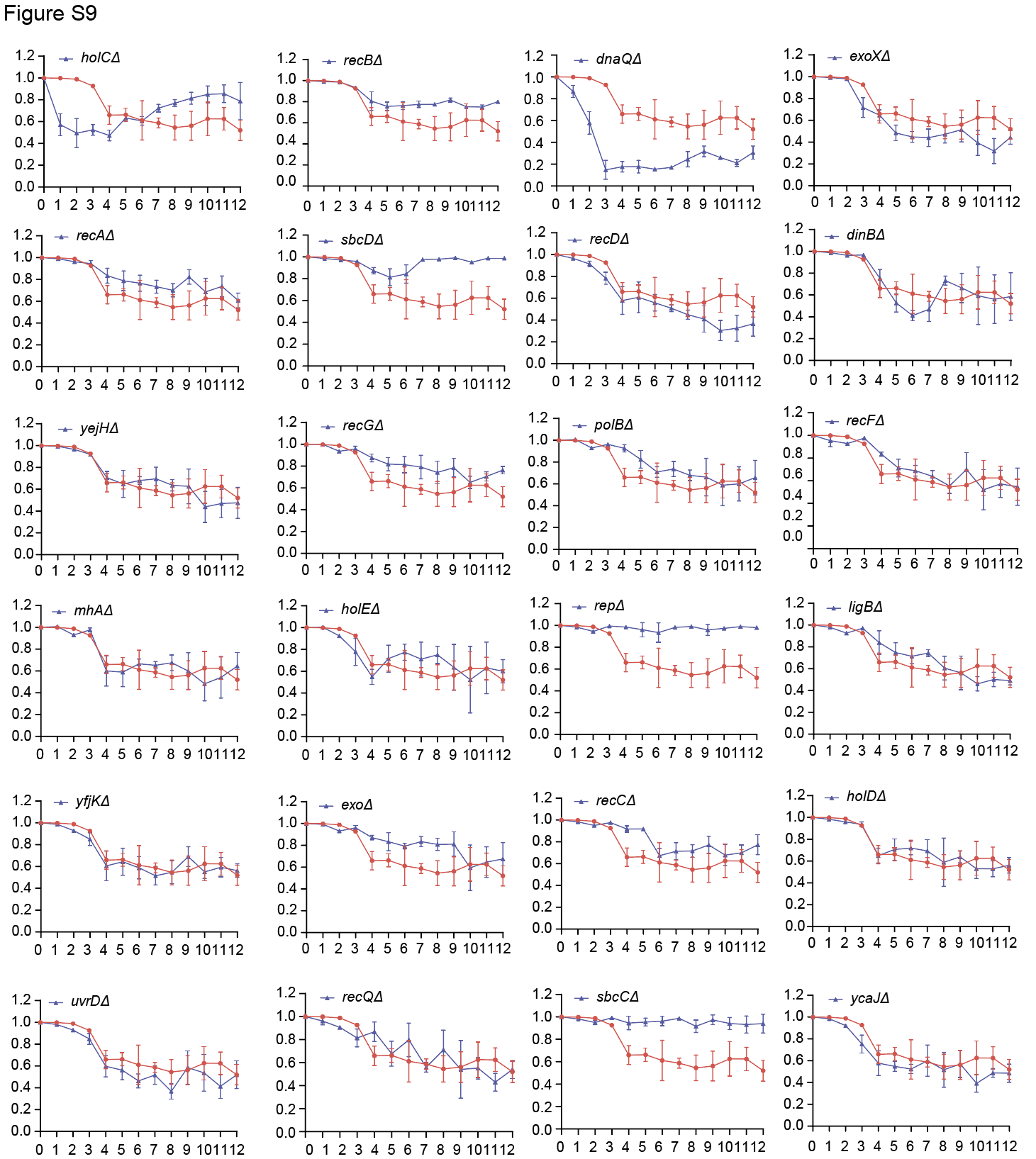


**Figure S9. Quantification of SIT structure repair in *E. coli* mutants compared with WT.**

Time-course quantification of SIT signal intensity in 24 E. coli mutants compared with the WT strain over a 12-day period following plasmid transformation. Each panel corresponds to a distinct mutant background, as indicated. SIT signal intensity in mutant strains is shown in red, while the corresponding WT signal is shown in blue. Signal intensities were normalized to the total signal and plotted as relative density. Data represents the mean ± standard deviation from three independent biological replicates.


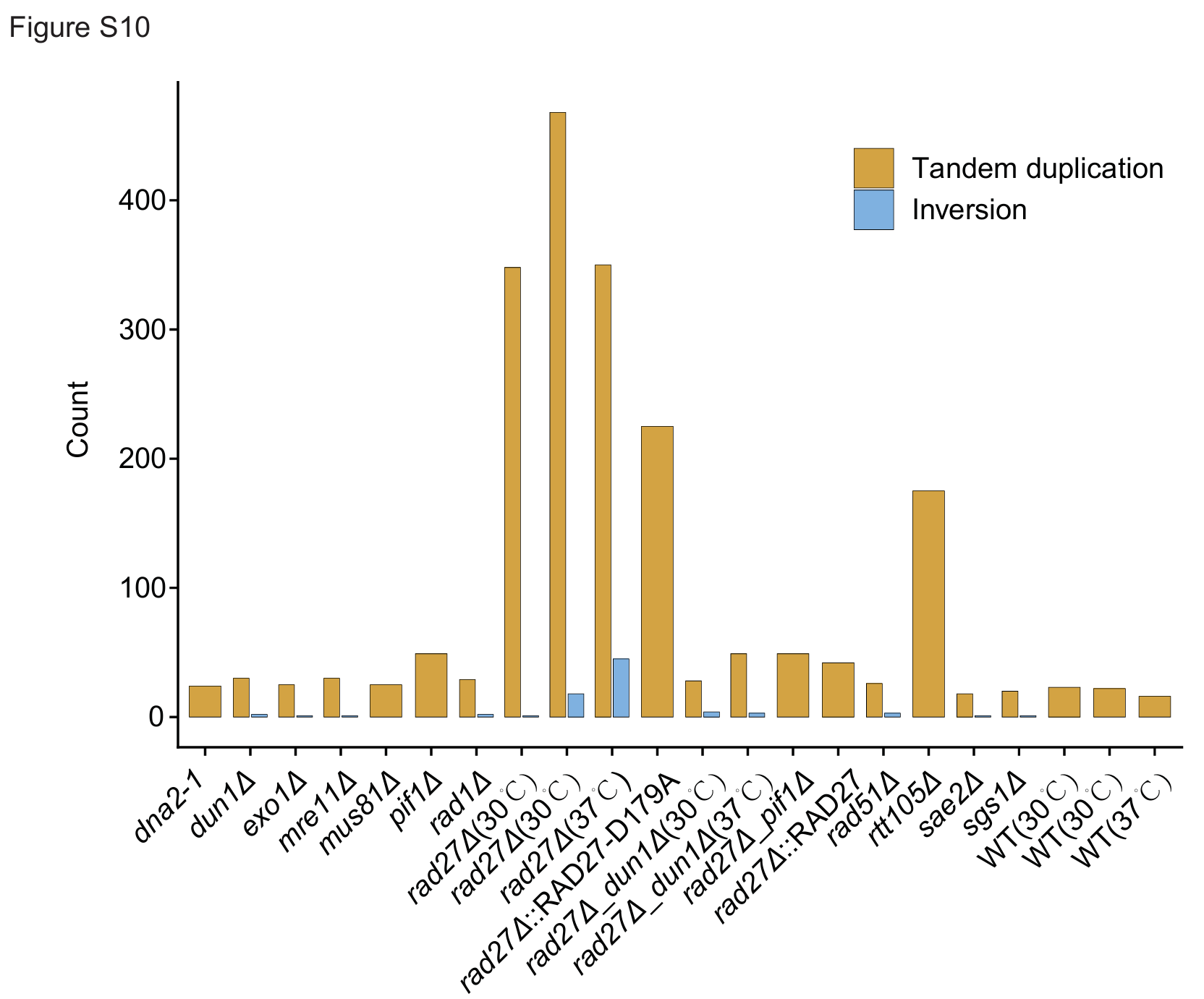


**Figure S10. Quantification of tandem duplications and inversions across yeast strains.**

Bar plots show the number of simple tandem duplication and inversion events detected in the indicated yeast strains using the PacBio long-read sequencing in this study. In wild-type strains grown at both 30 °C and 37 °C, simple duplications and inversions were detected at low background levels. The lose of Rad27 function exhibited a marked increase in both tandem duplications and inversions. Complementation with wild-type RAD27 largely reduced these events, whereas expression of the catalytic-dead RAD27-D179A mutant resulted in only partial suppression. Other mutants associated with elevated GCR rates showed only modest changes in simple duplications and inversions. These results indicate that loss of Rad27 preferentially promotes replication-associated rearrangements, providing a mechanistic context for the elevated SIT frequency observed in Rad27-deficient cells.


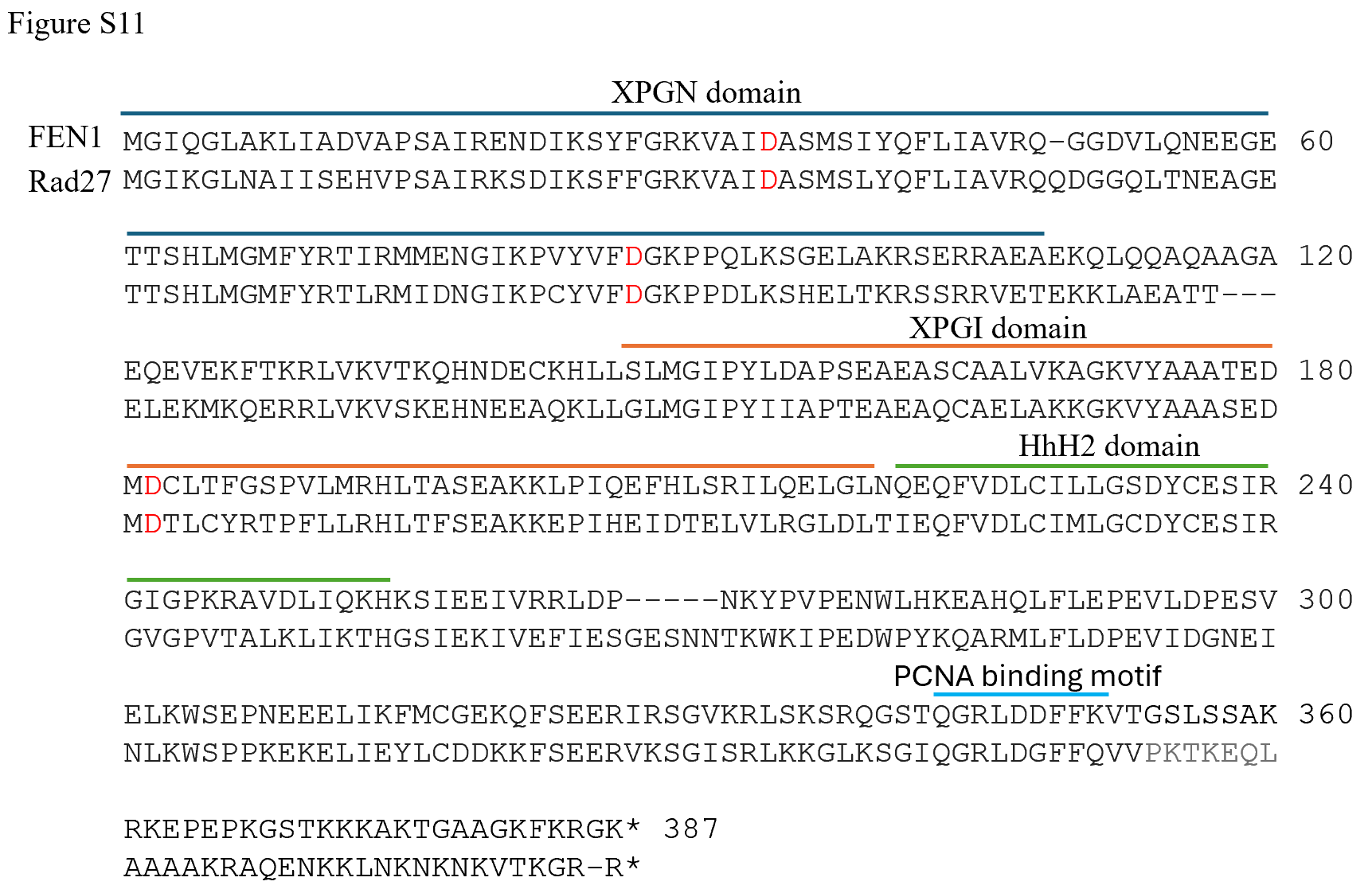


**Figure S11. Sequence alignment of human FEN1 and yeast Rad27 highlighting conserved functional domains.**
Protein sequence alignment between human FEN1 and its yeast homolog Rad27 shows strong conservation of key nuclease domains characteristic of the FEN1/XPG family, including the XPGN, XPGI, and helix–hairpin–helix (HhH2) domains involved in DNA binding and cleavage during Okazaki fragment processing, as well as the C-terminal PCNA-binding motif (PIP box). Conserved catalytic aspartate residues (D) are highlighted in red.


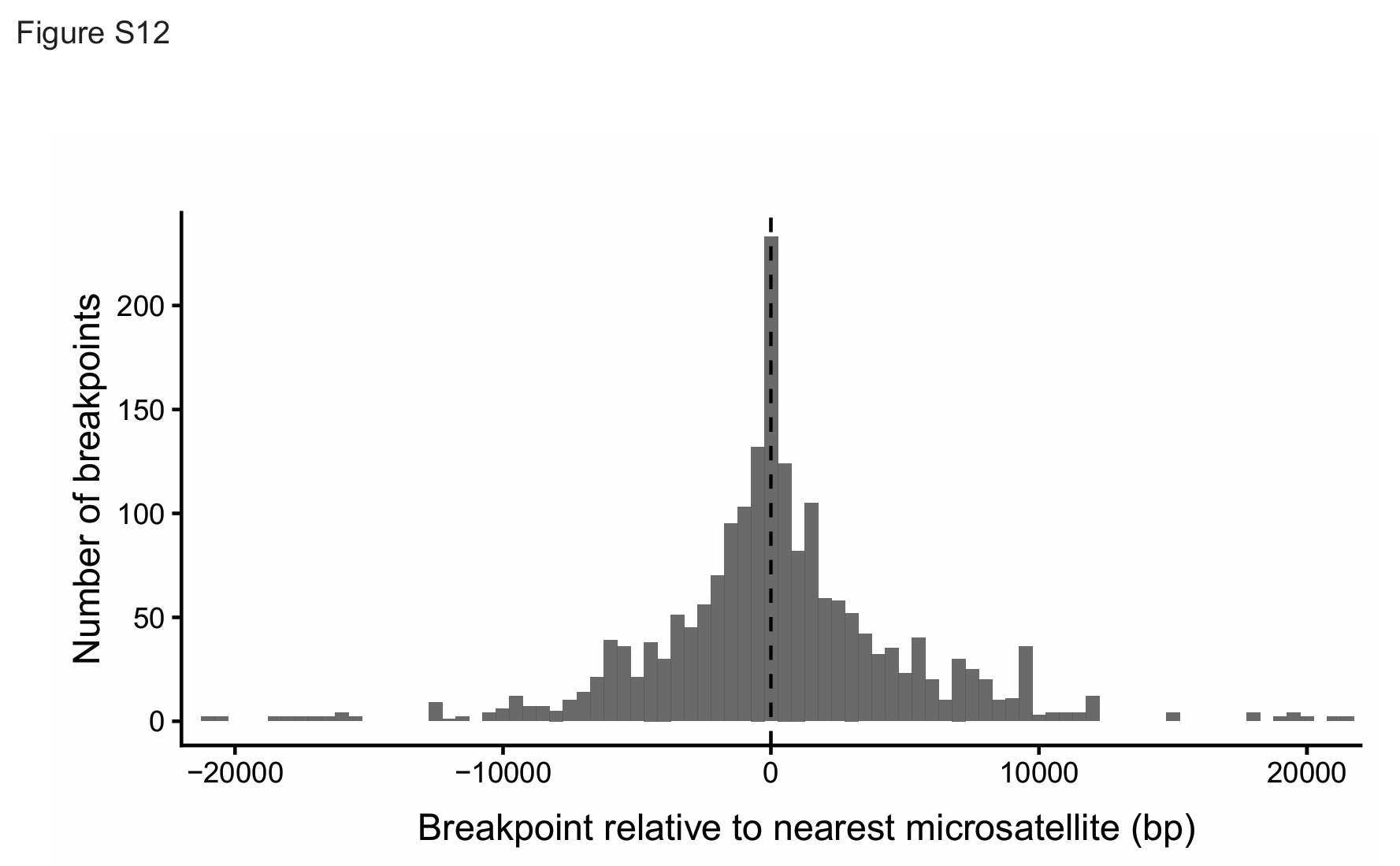


**Figure S12. Enrichment of SIT breakpoints near microsatellite DNA regions.**

Distribution of distances between SIT breakpoints and the nearest annotated microsatellite loci in the yeast genome. Microsatellites were identified using the Krait tool with default parameters. Distances were calculated as the genomic distance (bp) from each SIT breakpoint to its nearest microsatellite.
